# Silenced-C5ar1 improved multiple organ injury in sepsis rats via inhibiting neutrophil extracellular trap

**DOI:** 10.1007/s10735-023-10172-3

**Published:** 2024-01-02

**Authors:** Bin Shen, Qikai Shen, Qingqiu Zeng, Lingyan Zhang, Xiaofeng Li

**Affiliations:** 1 Department of Infectious Diseases, Huzhou Central Hospital, Huzhou, 313000 China; 2 Department of Intensive Care Units, Huzhou Central Hospital, Huzhou, 313000 China

**Keywords:** Sepsis, Complement C5a receptor 1, Transcriptome sequencing, Cecum ligation and puncture, Neutrophil extracellular traps

## Abstract

Sepsis has a systemic inflammatory response syndrome caused by infection. While neutrophils play contradictory roles in different stages of sepsis. Neutrophils have been proven to play an antibacterial role by producing neutrophil extracellular traps (NETs). Although the NET is beneficial to bacteria resistance, abnormal NET increases tissue damage. The complement C5a receptor 1 (C5ar1) is a gene related to strong inflammatory reactions and is found to be associated with inflammatory factors. This study found that there were 45 down-regulated genes and 704 up-regulated genes in sepsis rats by transcriptome sequencing. And those genes were significantly related to inflammation and immunity by GO and KEGG enrichment analysis involving the chemokine signaling pathway, the Toll-like receptor (TLR) signaling pathway, and the Fc gamma R-mediated phagocytosis. Additionally, the C5ar1 gene was significantly upregulated with interesting potential in sepsis and used for further study. This study used cecum ligation and puncture (CLP) rats that were respectively injected intravenously with PBS or the lentivirus vector to explore the effect of C5ar1 on CLP rats. It demonstrated that silenced- C5ar1 inhibited the ALT, AST, BUN, and CREA levels, improved the lung and spleen injury, and reduced the TNF-α, IL-6, IL-1β, IL-10, cf-DNA, and cfDNA/MPO levels. Additionally, silenced C5ar1 inhibited the TLR2, TLR4, and peptidylarginine deiminase 4 expression levels, which suggested the improvement of silenced C5ar1 on sepsis via inhibiting NETs and the TLR signaling pathway. This study provides a basis and new direction for the study of treatment on sepsis.

**Supplementary Information:**

The online version contains supplementary material available at 10.1007/s10735-023-10172-3.

## Introduction

According to a Global Burden of Diseases analysis, the sepsis mortality rate reduced by 52.8% between 1990 and 2017 as a result of the development of multiple organ support and other treatments, however, it is anticipated that sepsis mortality rate will still account for 19.7% of the global mortality (Rudd et al. 2020). Over the past two decades, timely use of antibiotics, fluid resuscitation, and multiple organ support therapies have gradually reduced sepsis mortality. But there is still sizable mortality and space for improvement. Sepsis, with high morbidity and mortality, has a systemic inflammatory response syndrome caused by infection (Du et al. 2022). Multiple organ dysfunction syndromes are characteristic in the later stage of sepsis and it’s hard to treat once it happens (Middleton et al. 2019). And in the innate immune system of sepsis, most congenital cells tend to experience apoptosis, such as dendritic cells, immature macrophages, and natural killer cells, while neutrophils show delayed apoptosis, which may be related to organ failure in late sepsis (Kovach and Standiford 2012). Ding et al. reported lung damage with neutrophil recruitment in sepsis rats (Ding et al. 2021). Neutrophils have been proven to play an antibacterial role by producing neutrophil extracellular traps (NETs), and NET formation is a process called NETosis (Tan et al. 2021). A study reported that although NET benefits bacteria resistance, abnormal NETs increased tissue damage (Colón et al. 2019). And degradation of NETs combined with antibiotic treatment can significantly improve the survival rate of septic mice (Czaikoski et al. 2016). Specifically, researchers found that degradation of NETs at 6 h after CLP improved organ damage (Mai et al. 2015). NETs were composed of extracellular chromatin decorated with histones and numerous granular proteins including myeloperoxidase (MPO) and elastase (NE) (Brinkmann and Zychlinsky 2007). Therefore, sepsis prevention and treatment need to explore the regulation of NETs in neutrophils. Although neutrophilic NETs are important in the treatment of sepsis, the research on their biological mechanisms is still insufficient.

In recent years, the technology of sequencing has been widely used in pathological and pharmacological studies of many diseases. New disease targets have been discovered through transcriptomics technology, and the new mechanisms will provide a basis and new direction for the study of disease diagnosis and treatment (Prokop et al. 2018). The potential diagnostic markers and therapeutic targets can be found through transcriptome sequencing to provide more exploration directions for disease diagnosis and treatment (Saeidian et al. 2020). Next-generation sequencing has become a first-line tool for the diagnosis of primary immunodeficiencies (Platt et al. 2021). And Riazuddin and co-workers identified 30 novel candidate genes for recessive intellectual disability by sequencing (Riazuddin et al. 2017). Especially, Middleton and co-workers used parallel techniques of RNA sequencing and ribosome footprint profiling to interrogate the platelet transcriptome and translatome in septic patients and healthy donors (Middleton et al. 2019). Therefore, this study explored the gene expression between sepsis rats with its control by transcriptome sequencing. And this study noticed the high expression of complement C5a receptor 1 (C5ar1).

C5ar1 is a gene related to strong inflammatory reactions and is found to be associated with interleukin (IL)-6, IL-1β, tumor necrosis factor (TNF)-α, and other inflammatory factors (Shi et al. 2021). A study found that inhibition of C5ar1 can antagonize NETs-induced thrombosis in vitro (Skendros et al. 2020). C5ar1 mice are more resistant to invasive meningococcal infection than wild-type mice, however, it is interesting that the pharmacological blockade of C5ar1 improves the survival rate of mice after sepsis induction (Herrmann et al. 2018). Additionally, in cancer cells, C5a induced the formation of ENTs in myeloid-derived suppressor cells (Ortiz-Espinosa et al. 2022). Because the biological mechanism of C5ar1 and ENTs of sepsis is still unclear, this study established an animal model of sepsis through cecal ligation and puncture (CLP) in rats, to explore the function of C5ar1, providing a basis and new direction for the study of diagnosis and treatment on sepsis patients.

## Materials and methods

### Construction of sepsis animal model

The sepsis animal model was constructed using the Sprague Dawley (SD) (200–230 g) rats with 7 days of adaptive feeding by cecum ligation and puncture (CLP) under specific pathogen-free conditions in the animal facility. The 36 SD rats were purchased from Shanghai Jihui Laboratory Animal Care Co. All procedures on animals are approved by the Animal Experimentation Ethics Committee of Zhejiang Eyong Pharmaceutical Research and Development Center (SYXK (Zhe)2021-0033). The isoflurane-anesthetized rats were made incisions in the middle of the abdominal wall to ligate at 1/3 from the end of the cecum by using the sterile No. 4 thread. Subsequently, a 21G injection needle was punctured about 3 to 4 times at the middle of the ligature site to the top of the caecum. The rats were sutured and resuscitated with 5 mL/100 g of normal saline. The sham rats underwent surgery like the CLP rats except for the operation of ligation and puncture. At 24 h after the CLP, whole blood was taken from 6 sham rats and 6 CLP rats and stored in the tube containing EDTA (BD Vacutainer, Franklin Lakes, NJ, USA) for RNA-seq. Then all rats were respectively injected intravenously with the PBS or the lentivirus vector (about 2 × 10^7^ transforming units) that contained the C5ar1 gene or the empty lentivirus vector. SD rats were divided into 4 groups (*n* = 6), the sham, the CLP, the sh-NC, and the sh-Serpinb1a groups. At 48 h after injection, blood was taken from rats of each group and was centrifugated for serum. After rats were euthanized, the lung and spleen were taken out and divided into two parts, one was fixed and embedded in paraffin, and the other was stored at − 80 °C.

### Transcriptome sequencing

The fresh blood samples were sent to LianChuang Biomedical Tech Co., Ltd, Hangzhou, China. The transcriptome sequencing was performed as Liao’s team reported (Liao et al. 2021). After quality evaluation of the total RNA by NanoDrop ND-1000 (NanoDrop, Wilmington, DE, USA), was detected by Bioanalyzer 2100 (Agilent, CA, USA). The mRNA was fragmented after specific capture by Dynabeads Oligo (25-61005, Thermo Fisher, USA). After normalizing the PCR libraries, the High throughput sequencing was started by Illumina Novaseq™ 6000 (LC Bio-Technology, China). After quality control, the raw data file (FASTQ) is used for subsequent analysis of differences and correlations. Log 2 (fold change) ≥ 1 was the gene threshold. The *P*-value < 0.05 is the standard for screening differential genes. Then Gene Ontology (Go) and Kyoto Encyclopedia of Genes and Genomes (KEGG) enrichment analyses were additionally performed on the genes.

### Histological analysis

The 5 µM paraffin-embedded sections of the lung and spleen were performed as described previously by Hematoxylin-eosin staining (HE) (Bry-001, Runnerbio, China) (Sun et al. 2021). All evaluations are semi-quantitative scores and are performed by professionals who were blinded to group allocation. The semi-quantitative assessment of pulmonary injury was scored on alveolar edema, alveolar septal thickening, and inflammatory cell infiltration (Wen et al. 2020; Yang et al. 2021). And spleen injury was scored on histomorphology and the area of red pulp and white pulp, and edema (Zhang et al. 2019). Semi-quantitative scores were determined by two independent observers unaware in a blind fashion.

### Quantitative determination of cell free-DNA (cf-DNA) and the cf-DNA bound to MPO (cf-DNA/MPO)

To detect NETs, the cf-DNA and cf-DNA/MPO levels were measured as previously described (Czaikoski et al. 2016). The PicoGreen dsDNA kit (P7589, Invitrogen, USA) was used to measure the level of the cf-DNA in serum. In brief, at room temperature, the serum diluted with PBS (1:10) and the fluorescent reagent were mixed in a 96-well plate and the absorbance were detected by the plate reader at the wavelength of 485/538 nm. The cf-DNA concentration was calculated according to the standard curve. In addition, the concentration of cf-DNA/MPO was measured in the 96-well plate that coated with anti-MPO monoclonal antibody (ab208670, Abcam, UK) using the PicoGreen dsDNA kit as above.

### Inflammatory cytokines level analysis

The inflammatory cytokines were measured by enzyme-linked immunosorbent assays (ELISA) according to the instructions. The interleukin 6 (IL-6) (MM-0190R2, MEIMIAN, China), IL-10 (PI525, Beyotime, China), IL-1β (MM-0099R2, MEIMIAN, China), and TNF-α (MM-0180R2, MEIMIAN, China) were used for evaluating the level of inflammation in serum. The OD was measured at 450 nm by a microplate reader (CMaxPlus, Molecular Devices, USA). Their concentration was directly proportional to the OD (450 nm) value, which was calculated by plotting a standard curve.

### Semi-quantitative analysis of protein levels

Western blot was used to perform the semi-quantitative analysis of the proteins in the neutrophils. Neutrophils were isolated from rat blood using Percoll solution (P8370, Solarbio, China) by density gradient centrifugation (30 min at 500×*g*) as previously reported (Xue et al. 2017). Neutrophils were resuspended in RPMI-1640 containing 5% FBS. Then after lysing of the neutrophils by RIPA lysis buffer (P0013E, Beyotime, China) with protease and phosphatase inhibitors (P0013C, Beyotime, China), the quantitative analysis of total protein levels was measured using a BCA kit (4240GR100, Beyotime, China) in 96-well plate. The denatured proteins were separated in SDS-PAGE electrophoresis, transferred to the PVDF membrane, and incubated with 5% fat-free milk. Subsequently, the blocked membranes were handled at 4 °C with anti-TLR2 (1:2000, 17236-1-AP, Proteintech, USA), anti-TLR4 (1:2000, AF7017, Affinity, USA), anti-peptidylarginine deiminase 4 (PAD4) (1:5000, DF6685, Affinity, USA) and anti-C5ar1 (1:5000, DF10210, Affinity, USA), and anti-GAPDH (1:30000, AF7021, Affinity, USA) antibodies overnight and further incubated with corresponding secondary antibody (1:5000, CST, USA) at RT for 1.5 h. Finally, the membrane was incubated with ECL reagents (610020-9Q, Qing Xiang, China) and visualized by ImageJ software.

### Statistical analysis

The multiple group’s data with normal distribution and the homogeneous variance were analyzed by the One-way-ANOAY following the Tukey test. And the Kruskal–Wallis *H* test was used to analyze the data without the normal distribution. All data were analyzed by SPSS 26.0. and expressed as mean (standard deviation), *P* < 0.05 means the difference was statistically significant.

## Results

### Screening and analysis of potential target genes

**Fig. 1:**
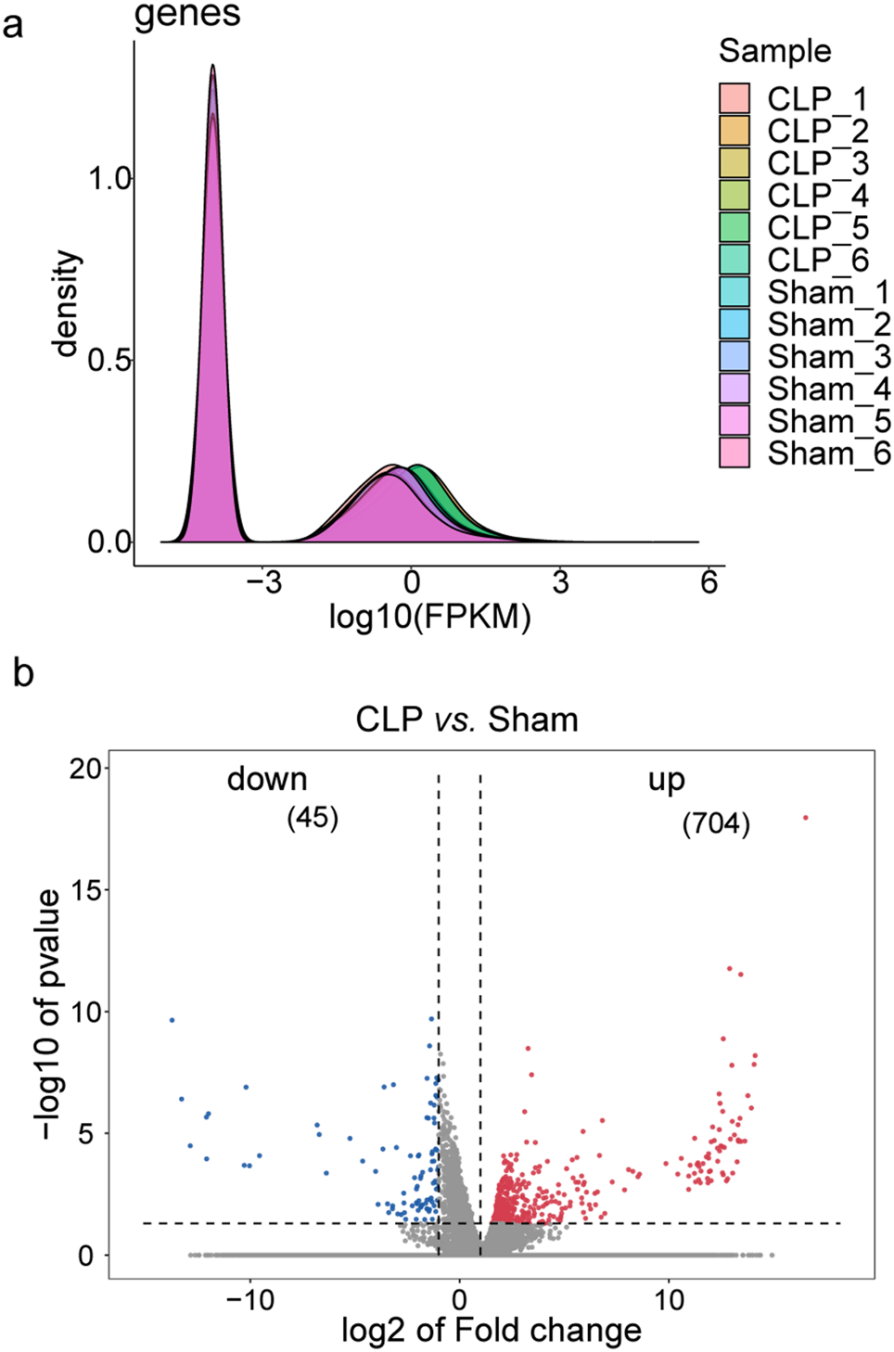
Gene expression value density and differentially expressed genes overall distribution. **a** The expression value density diagram. **b** The volcano of scatters plots. The red represents significantly up-regulated genes, blue represents significantly down-regulated genes, and gray represents the gene expression without significant differential

**Fig. 2:**
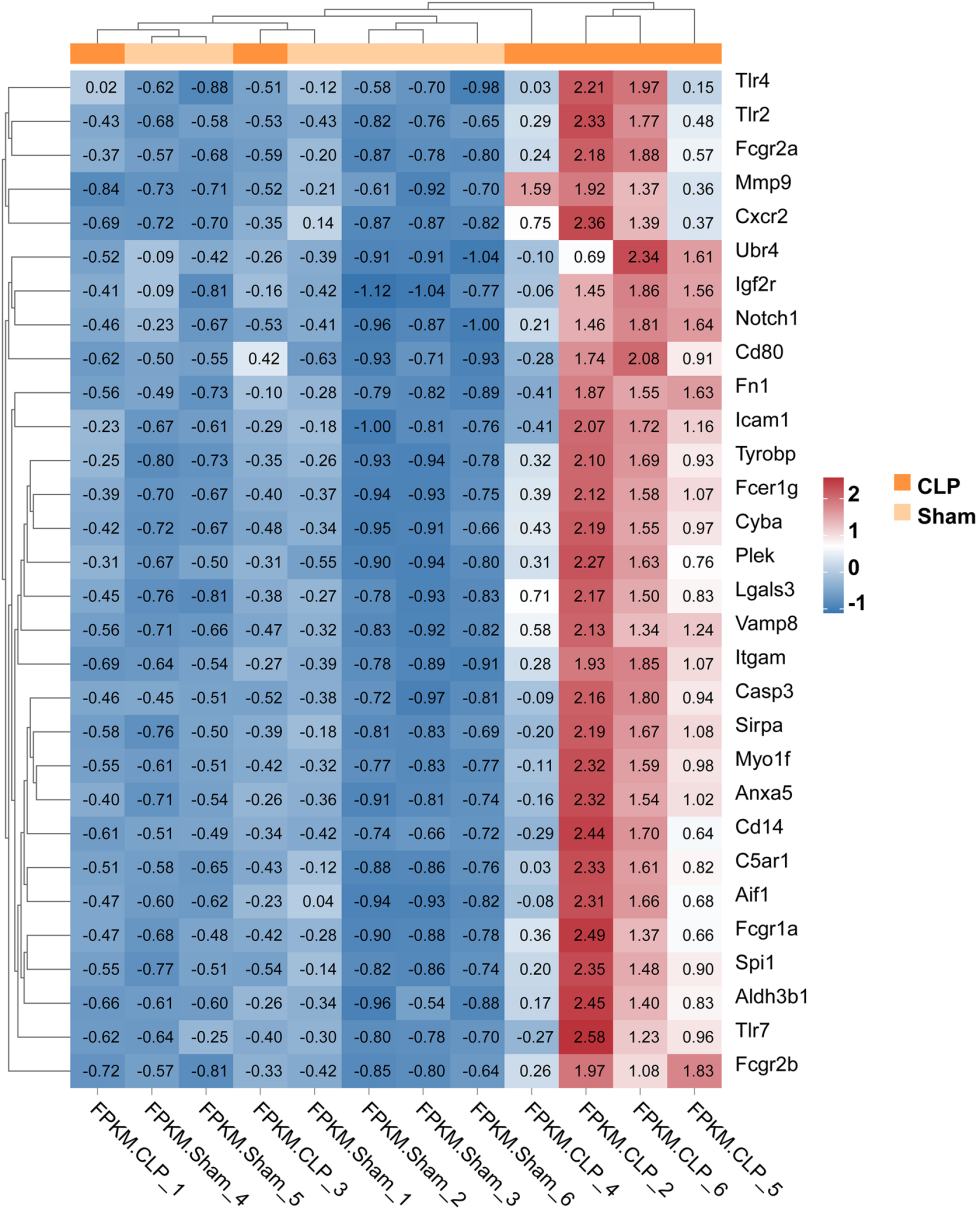
A heatmap of FPKM values for top 30 genes that ranked by number of neighbors. Each row of values was homogenized. Red indicates high expression, blue indicates low expression, and white indicates no difference

Comparative analysis of CLP and sham rat genes by transcriptome sequencing. The expression trend of biological repeated samples tends to be consistent (Fig. 1a). And the volcanic map shows the overall distribution of differentially expressed genes, there are a total of 32,623 genes, 704 up-regulated genes, and 45 down-regulated genes (Fig. 1b). The Heatmap was generated by cluster analysis of the Z value for all significantly different genes (Supplementary Fig. 1) and showed the top 100. Additionally, we performed the PPI network construction and analysis for all significantly upregulated genes by STRING. The PPI was modified by the Cytoscape to remove neighbor-free nodes (Supplementary Fig. 2). An average number of node degree is 9.025, which means there are relevant number of neighbors. There are 189 genes with number of neighbors greater than 9.025. The Supplemental Table 1 lists 183 gene with neighbor degrees greater than average that can find the ENSEMB ID in DAVID database, including Fn1 (degree = 67), Tlr4 (degree = 64), Mmp9 (degree = 62), Tyrobp (degree = 62), Tlr2 (degree = 58), Itgam (degree = 54), Vamp8 (degree = 49), Casp3 (degree = 47), C5ar1 (degree = 46), et al. The expression of top 30 of these 183 genes were shown in Fig. 2.

### Enrichment analysis

**Fig. 3:**
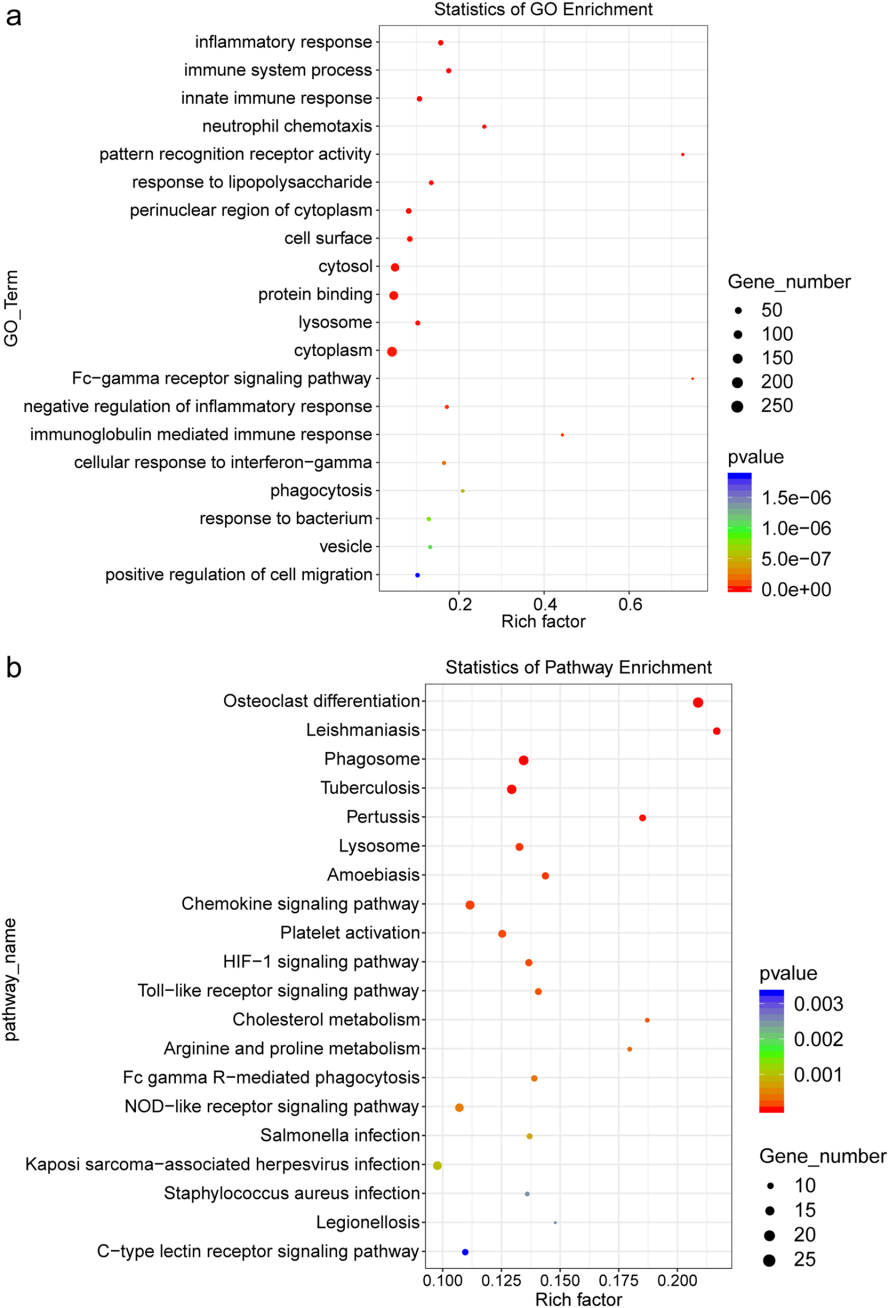
The enrichment analysis of Protein-Protein Interaction (PPI) networks. **a** The bubble chart of Gene Ontology (GO) enrichment for all potential target genes. There are shown the top 20, and the rich factor means that in the GO term, the ratio of the number of differential genes located to the number of total genes located. **b** The bubble chart of KEGG enrichment for all potential target genes. There are shown the top 20 pathways with the lowest *P*-value. The Rich Factor indicates the ratio of the potential target gene number in the pathway to the total gene number in the pathway, the value of the Rich Factor is proportional to the enrichment degree

Additionally, the results of the GO enrichment analysis of 704 up-regulated genes showed that the inflammatory response, the immune system process, the innate immune response, and the neutrophil chemotaxis were the top 4 terms with the lowest *P*-value (Fig. 3a and d) showed the statistics of KEGG pathway enrichment and most of those pathways are involved in immunity and inflammation, such as the chemokine signaling pathway, the Toll-like receptor signaling pathway, and the Fc gamma R-mediated phagocytosis.

**Fig. 4:**
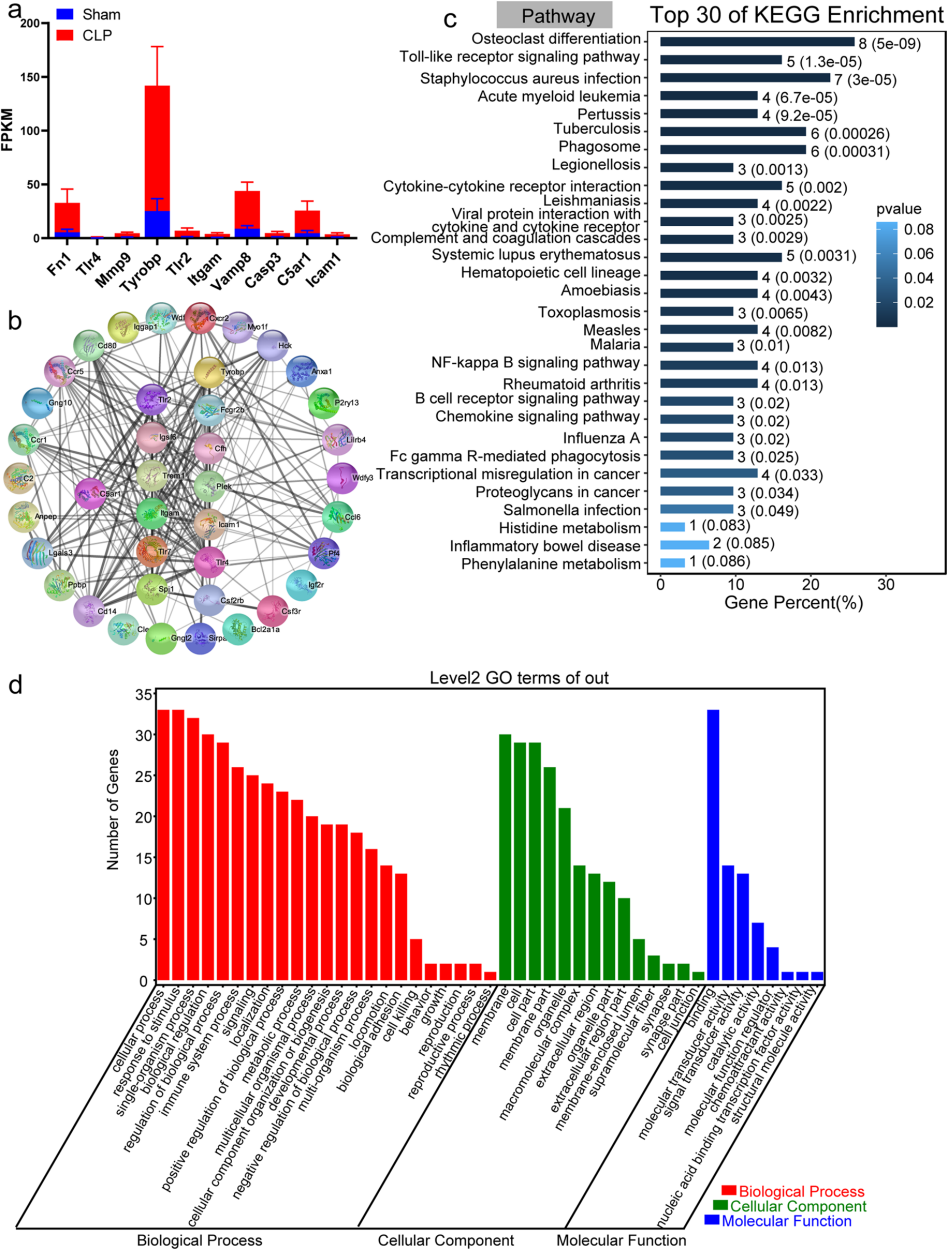
Enrichment analysis of complement C5a receptor 1 (C5ar1) and its neighbors. **a** The expression levels of Fn1, Tlr4, Mmp9, Tyrobp, Tlr2, Itgam, Vamp8, Casp3, C5ar1, and Icam1 in CLP rats. **b** Protein–Protein Interaction (PPI) networks of C5ar1 and its neighbors. **c** The bar plot of top 30 pathways from KEGG enrichment for C5ar1 and its neighbors. **d** The bar plot of the top 30 Gene Ontology (GO) terms in the class of biological process, cellular component, and molecular function. The GO enrichment analysis was performed on C5ar1 and its neighbors

Accordingly, we observed the expression levels of top 10 genes in Supplemental Tables 1 and found the expression levels of Fn1, Tyrobp, Vamp8, and C5ar1 were enormously increased in CLP rats (Fig. 4a). We have noticed that C5ar1 related proteins include TLR2, TLR4, Casp3, etc. (Fig. 4b). The 47 genes that related to C5ar1 were enriched and analyzed using GO and KEGG enrichment analysis function on the GENE DENOVO platform (http://www.omicshare.com), and the results are shown in Fig. 4c, d. They were mainly involved in the Osteoclast differentiation, the Staphylococcus aureus infection, the Tuberculosis, the Phagosome, the Toll-like receptor signaling pathway, and the Cytokine-cytokine receptor interaction pathways (Fig. 4c). Especially, the main biological processes involved include immune system process, response to stimulus and multi-organism process (Fig. 4d).

### The amelioration of silenced-C5ar1 in CLP rats on liver, kidney, lung, and spleen injury

**Fig. 5:**
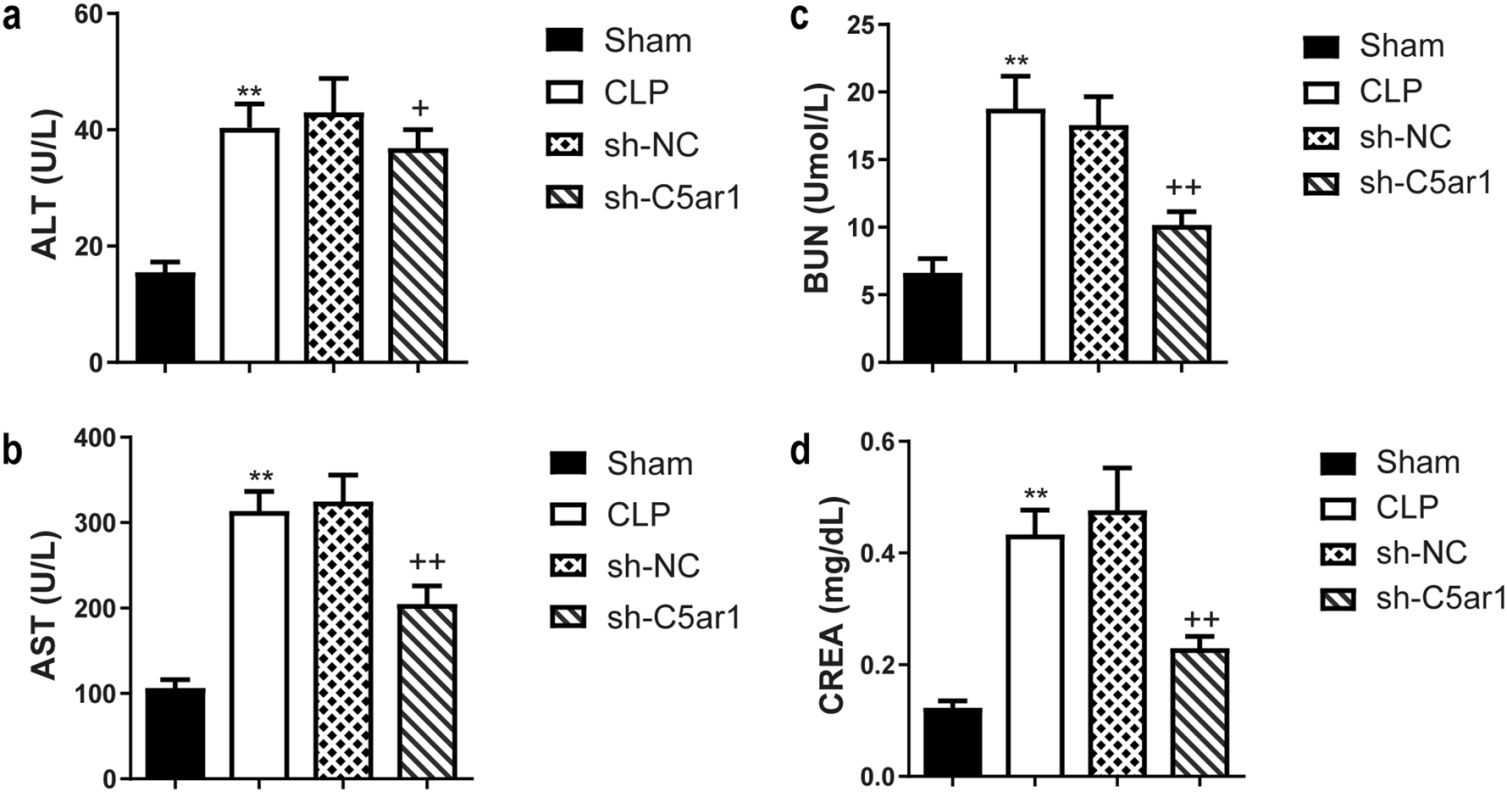
Blood biochemical index of sepsis rats. **a** ALT and **b** AST indicate the degree of liver damage. **c** BUN and **d** CREA reflect the degree of renal injury. Data are presented as mean (standard deviation), *n* = 5. ^**^P <0.01, compared to the sham group; ^+^*P* <0.05 and ^++^*P* <0.01, compared to the sh-NC group. *ALT* alanine aminotransferase, *AST* aspartate aminotransferase, *BUN* blood urea nitrogen, *CREA* creatinine

ALT, AST, BUN, and CREA, as biochemical indexes, were used to evaluate the severity of liver and kidney (Fig. 5a–d). The values of ALT, AST, BUN, and CREA were dramatically increased in CLP rats (*P* <0.01). Additionally, silenced-C5ar1 markedly inhibited the ALT, AST, BUN, and CREA levels (*P* <0.05 and *P* <0.01) (Fig. 5a–d).

**Fig. 6:**
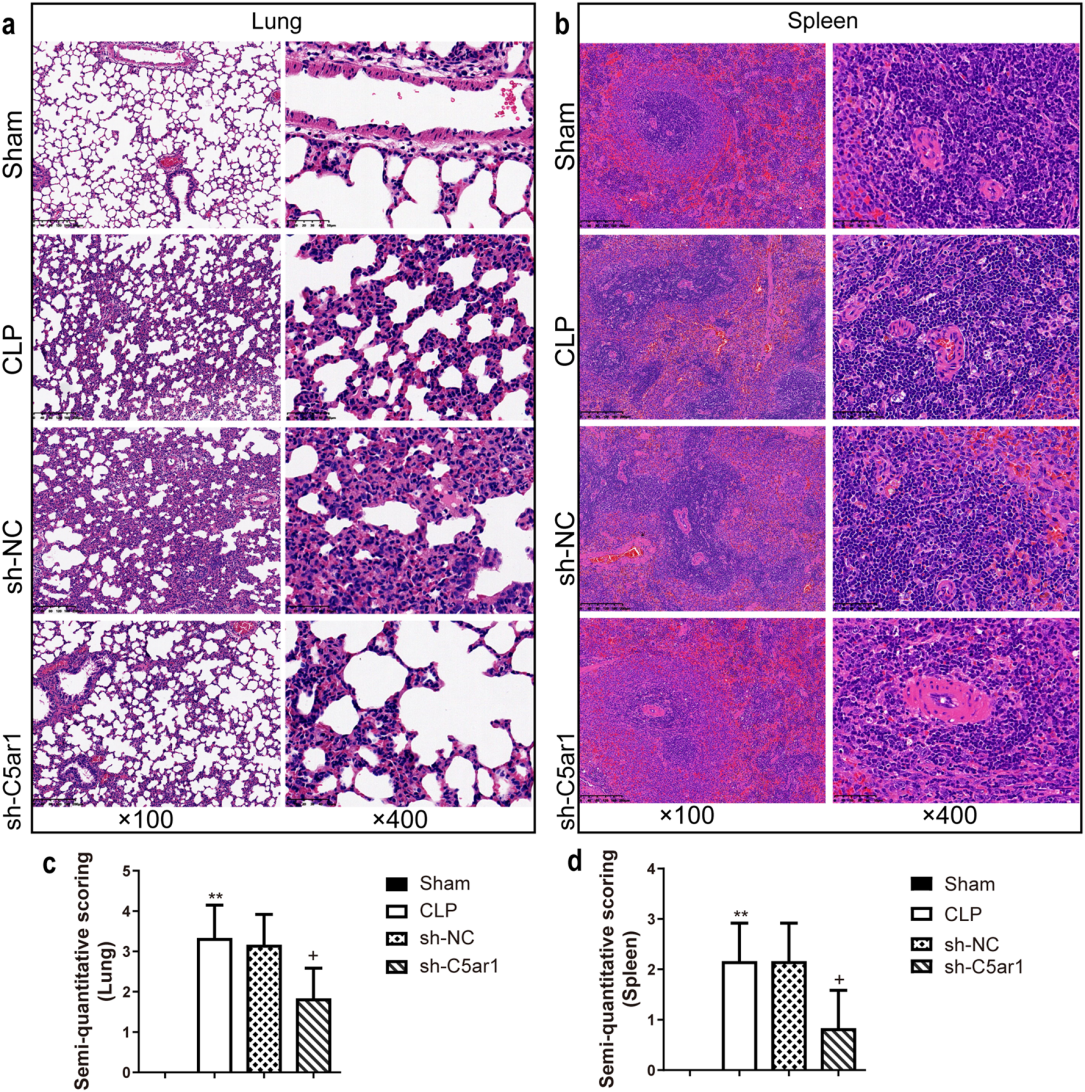
Histological damage in sepsis rats by using Hematoxylin-eosin staining. **a** The representative images of the lung. **b** The representative images of the spleen. And the semi-quantitative scoring of the lung **c** and **d** by professionals who were blinded to group allocation. Magnification, ×100 and ×400 and scale bar = 50 μm; data are presented as mean (standard deviation), *n* = 5. ^**^P <0.01, compared to the sham group; ^+^*P* <0.05, compared to the sh-NC group

The HE staining was used to evaluate the lung and spleen injury degree (Fig. 6). In the sham group, the lungs had complete alveolar structure without neutrophil infiltration or edema, while in the CLP and sh-NC groups, the lungs had thickened alveolar wall, damaged alveolar structure, and infiltrated inflammatory cells which were improved by silenced-C5ar1 (Fig. 6a). In sham rats, the spleen had a complete structure and a clear dividing line between the red and white medulla, while the dividing line was blurred, the white medulla area was expanded, and the red medulla was shrunk in the CLP and sh-NC groups (Fig. 6b). And in the sh-C5ar1 group, the dividing line between the red and white medulla was clearer compared to the sh-NC group (Fig. 6b). The lung and spleen semi-quantitative scores of the CLP group were dramatically larger than that of the sham group (*P* <0.01) (Fig. 6c, d), while their semi-quantitative scores were decreased by silenced-C5ar1 treatment (*P* <0.05) (Fig. 6c, d).

### The amelioration of silenced-C5ar1 in CLP rats on inflammatory cytokines and NET

**Fig. 7:**
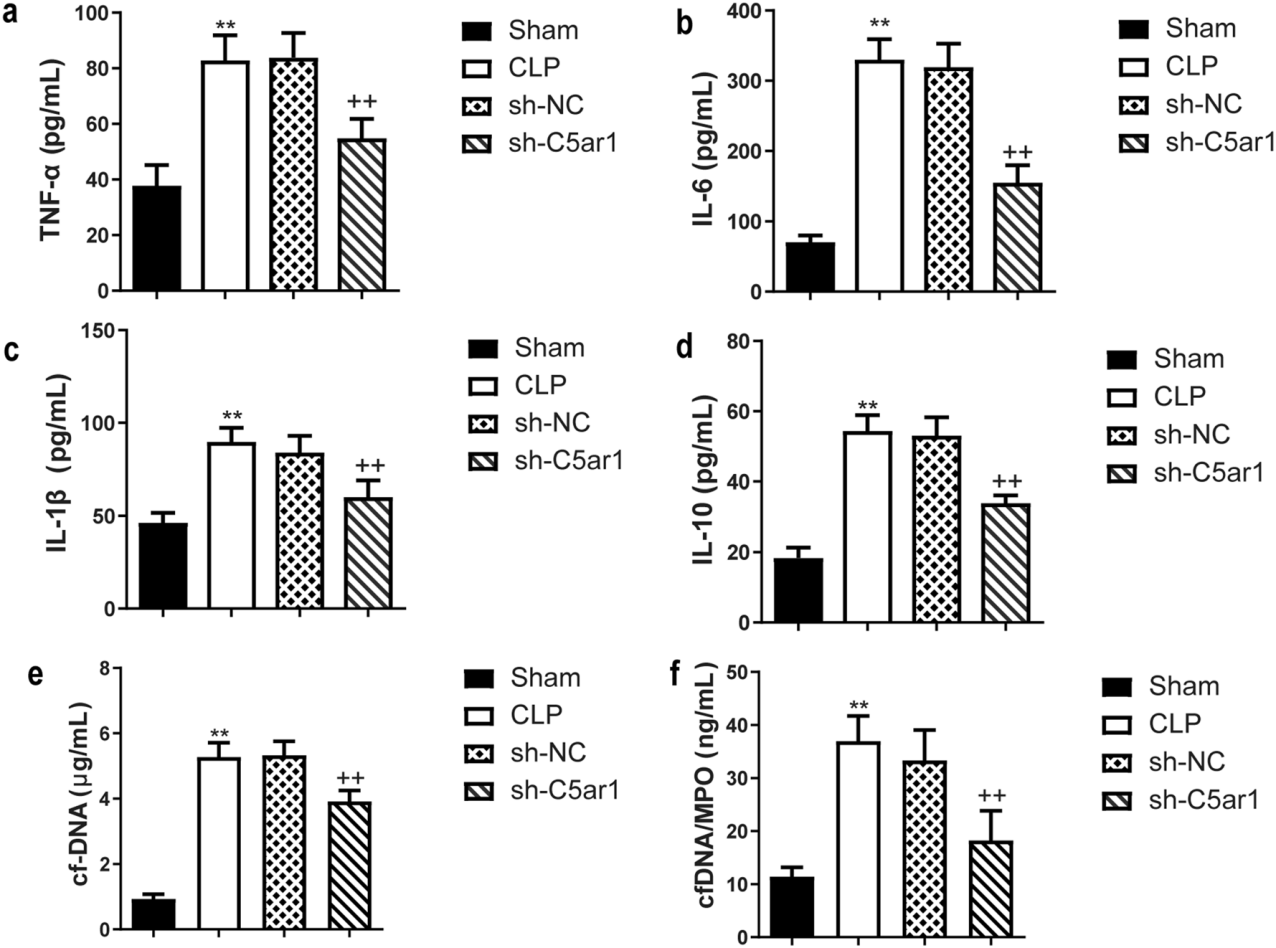
The inflammatory cytokine, cf-DNA and cf-DNA/MPO levels in serum of CLP rats. The **a** TNF-α, **b** IL-6, **c** IL-1β, and **d** IL-10 levels were measured by enzyme-linked immunosorbent assays. The **e** cf-DNA and **f** cf-DNA/MPO were measured by a PicoGreen dsDNA kit to observe the NETs levels. Data are presented as mean (standard deviation), *n* = 5. ^**^P <0.01, compared to the sham group; ^++^*P* <0.01, compared to the sh-NC group. *Interleukin* IL-6, *TNF* tumor necrosis factor, *cf-DNA* cell free

The ELISA was used to measure the TNF-α, IL-1β, IL-6, and IL-10 levels. Those levels were increased in the CLP rats, while they were significantly decreased in the silenced-C5ar1 rats in comparison with the sh-NC rats (*P* <0.01) (Fig. 7a–d). The cf-DNA and cf-DNA/MPO is major constituents of NET (Czaikoski et al. 2016). As shown in Fig. 7e, f, the cf-DNA and cf-DNA/MPO were huge increased in CLP rats compared to sham rats (*P* <0.01), while they were dramatically inhibited in silenced-C5ar1 treatment on CLP rats (*P* <0.01).

### The levels of TLR2, TLR4, PAD4, and C5ar1 in neutrophils of CLP rats

**Fig. 8:**
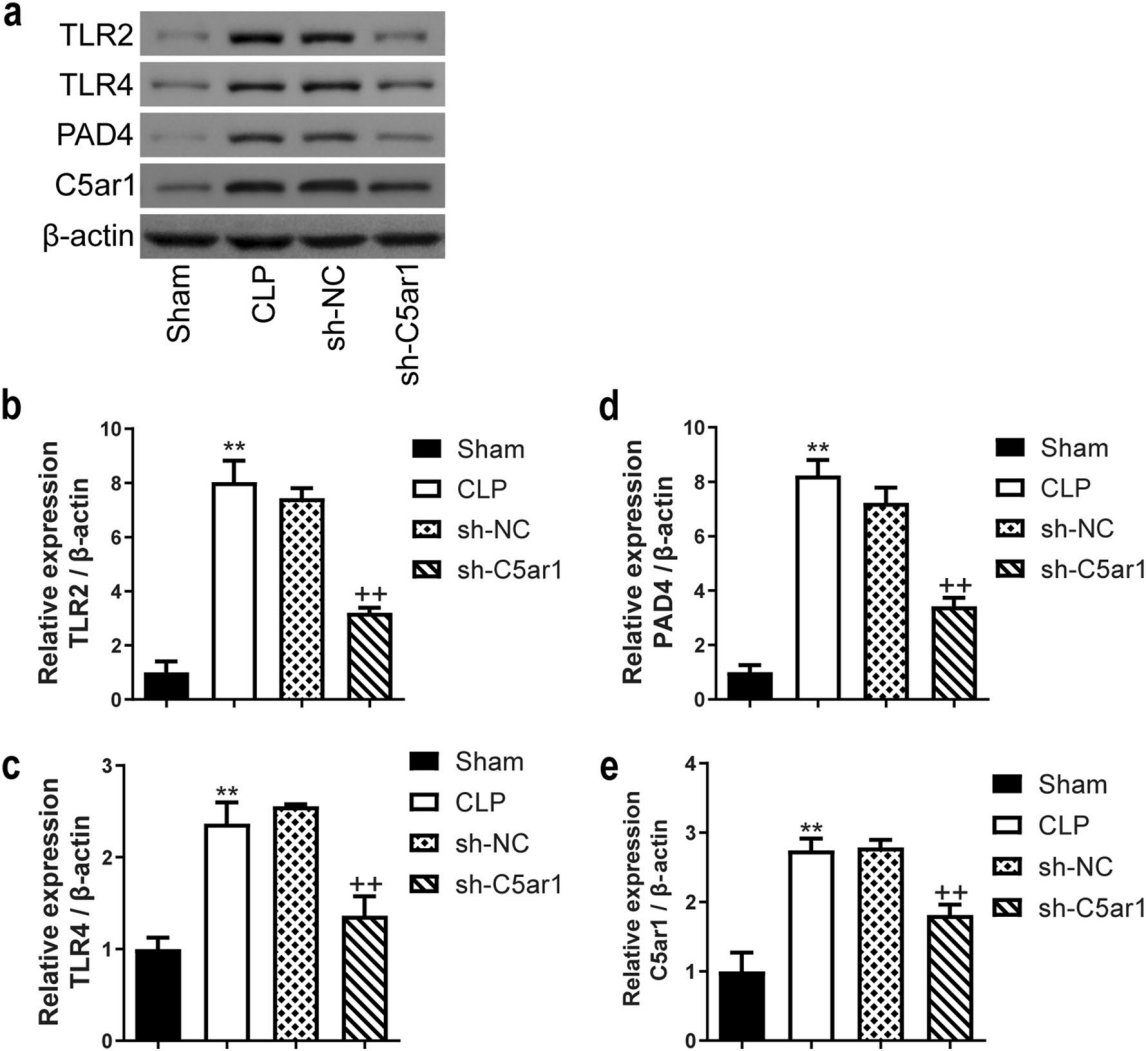
The expression levels of TLR2, TLR4, PAD4, and C5ar1 of peripheral blood neutrophils in CLP rats. **a** The protein bands of TLR2, TLR4, PAD4, and C5ar1; **b**–**e** the results of relative expression analysis. Data are presented as mean (standard deviation), *n* = 3. ^*^P <0.05 and ^**^P <0.01, compared to the sham group; ^++^*P* <0.01, compared to the sh-NC group. *TLR* toll-like receptor, *PAD4* peptidylarginine deiminase 4, *C5ar1* complement C5a receptor 1

The Western Blot was used to measure the TLR2, TLR4, PAD4, and C5ar1 levels in neutrophils (Fig. 8a). Semi-quantitative analysis of the protein bands revealed the TLR2, TLR4, PAD4, and C5ar1 levels were significantly increased in CLP rats with the sham rats as control (*P* < 0.05 and *P* < 0.01), in the contrary, the TLR2, TLR4, PAD4, and C5ar1 levels were significantly decreased in silenced-C5ar1 treatment on CLP rats (*P* < 0.01) (Fig. 8b–e).

## Discussion

In this study, the gene different between CLP rats and control rats were observed by transcriptome sequencing. There are a total of 32,623 genes, 704 up-regulated genes, and 45 down-regulated genes. Inflammation - and immunity related GO terms had the most significant differences among the results of GO enrichment for all differential genes, in which C5ar1 were related to the inflammatory response, Response to lipopolysaccharide, apoptotic process, and neutrophil chemotaxis. Additionally, this study found that silenced C5ar1 could improve tissue injury, inhibit inflammation. After CLP 24 h, detecting the ALT, AST, BUN, and CREA levels, this study found that rats had injury of liver and kidney function, also, the HE staining observed the damage of lung and spleen in CLP rats. Interestingly, silenced-C5ar1 could improve those damages suggesting high expression of C5ar1 play a crucial role in the damages of tissue in sepsis. This may be related to the regulate of inflammatory factors and anti-inflammatory cytokines after silenced C5ar1. In this study, the IL-6, TNF-α, IL-1β, and IL-10 levels were all reduced. It is well known that increased IL-6 levels in sepsis patients are associated with increased mortality, and a study reported that decreasing IL-6 levels can reduce organ failure in sepsis patients (Liu et al. 2021; Panacek et al. 2004). Also, a study reported that a lower IL-6 level was beneficial to sepsis (Riedemann et al. 2003). Furthermore, in the late stage of sepsis, the anti-inflammatory reaction is dominant, and the increase of IL-10 level can predict the mortality of severe sepsis (Monneret et al. 2004). To sum up, this study demonstrated that silenced-C5ar1 could improve the tissue damage and inflammatory homeostasis of septic rats.

Studies have shown that C5 inhibitors are anti-inflammatory in COVID-19 critically ill patients and decreased neutrophil counts and attenuated NET release (Mastellos et al. 2020). Furthermore, studies have found that sepsis has complex proinflammatory and anti-inflammatory responses, and the destruction of immune balance leads to organ dysfunction and lethality (Delano and Ward 2016). Especially, neutrophils are the key cells of the body against bacterial infection and have a strong antibacterial effect. Study reported that although neutrophils are very important to eliminate bacteria in sepsis, their excessive infiltration will also promote organ failure in the late stage of sepsis. Persistent recruitment of neutrophils and delayed apoptosis may be the main cause of organ failure (Bhan et al. 2016). NET, as the key mechanism of neutrophil antibacterial (Janicova and Relja 2021), scientists have found that its dysregulation increases sepsis associated liver tissue damage(Bukong et al. 2018). It suggests that homeostasis of NET is very important to improve the level of sepsis tissue damage and inflammation. Modern research showed that generation of C5a is association with inflammation in sepsis (Zetoune and Ward 2020). Silencing C5aR1 increased survival of CLP mice in sepsis and decreased the secretion of cytokines (Muenstermann et al. 2019; Kalbitz et al. 2016). Additionally, a study reported that C5a induced the change in polarization of neutrophils (Denk et al. 2017). Polarization of neutrophils to the N2 type exhibits a longer lifespan and releases IL-6 and NET to promote cancer development (Wang et al. 2018). Moreover, C5aR1-knock-down animals has a lower MPO level in lung of mice with polytrauma and hemorrhagic shock (Chakraborty et al. 2021). Additionally, the PAD4 is a crux of the NET formation (Hawez et al. 2022). This study found silenced-C5ar1 inhibited the PAD4 level in neutrophils. Although C5a activation of neutrophils has been shown, association of C5ar1 with NETs is less clear. This study investigated the relationship between C5ar1 and NET levels in neutrophils from sepsis rat by silencing C5ar1 in CLP rats and demonstrated the effect of silenced-C5ar1 to reduce NET levels.

In this study, 189 potential key genes were screened, of which the top 10 genes were related to inflammation and anti-inflammatory reaction. Additionally, the KEGG enrichment analysis of C5ar1 and its related genes showed that Toll-like receptor signaling pathway may be related to these genes. Moreover, this study predicted interactions of TLR2, TLR4 and C5ar1. Research have shown that NET can promote the differentiation directly via TLR2 (Wilson et al. 2022) suggesting silenced-C5ar1 inhibited NET to reduce the Tlr2 level. While song et al. reported that extracellular vesicles can promote CLEC5A and TLR2 levels of neutrophils and macrophages, thereby induce NET formation (Sung et al. 2019). This study shown that silenced-C5ar1 inhibited the degree of NET and TLR2 suggesting silenced-C5ar1 may improve the sepsis via the Toll-like receptor signaling pathway. The causal relationship between them needs to be further verified. This study provided a new idea and research direction for the treatment of sepsis.

In conclusion, through transcriptome sequencing, this study reported that there were 704 up-regulated genes and 45 down-regulated genes in CLP rats compared to sham rats. Enrichment analysis showed that these genes are involved in immune, inflammatory, and infection-elated response and signaling pathways. Moreover, this study found C5ar1 has a high expression in CLP rats and predicted that C5ar1 may be related with the TLR signaling pathway. Additionally, this study found silenced C5ar1 in CLP rats inhibited the levels of ALT, AST, BUN, CREA, TNF-α, IL-6, IL-1β, IL-10, cf-DNA, and cf-DNA/MPO in serum, and decreased the expression levels of TLR2, TLR4, and PAD4 proteins in neutrophils. Moreover, silenced-C5ar1 improved lung and spleen damage of CLP rats. Summing up, this study suggested that inhibiting the C5ar1 level can inhibit over-expression ENTs to balance the inflammatory levels in CLP rats and improve the tissue damage in sepsis, providing a basis and new direction for the study of treatment on sepsis patients.

### Supplementary Information

Below is the link to the electronic supplementary material.

Supplementary material 1 (PDF 607.9 kb)

The information of key genes from PPI analysis. Supplementary material 2 (XLSX 17.6 kb)
