# Supplementary material for: Silenced-C5ar1 improved multiple organ injury in sepsis rats via inhibiting neutrophil extracellular trap
Source: J Mol Histol. 2024 Jan 2;55(1):69–81. doi: 10.1007/s10735-023-10172-3 (PMC10830609; doi:10.1007/s10735-023-10172-3)
Supplement: Supplementary file 1 — Supplementary material 1 (PDF 607.9 kb) [file 10735_2023_10172_MOESM1_ESM.pdf]

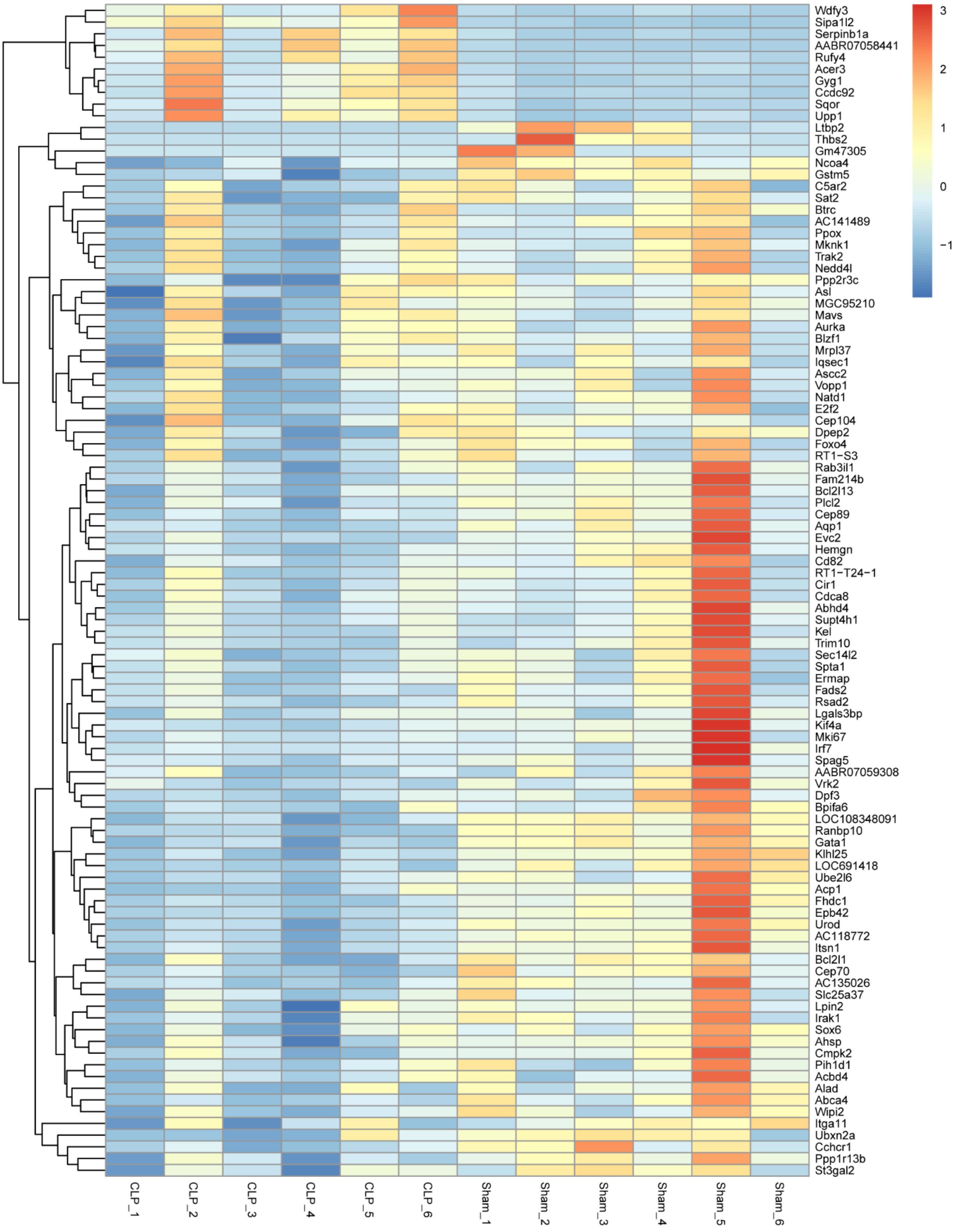

Supplemental Figure 1: Heatmap of all significantly different genes. It was generated by cluster analysis of the Z value. There are shown the Top 100 with the lowest *P*-value according to the value of log10 (FPKM + 1). Color from blue via white to red indicates the gene expression from low to high. Genes are sorted from small to large according to the *P*-value.

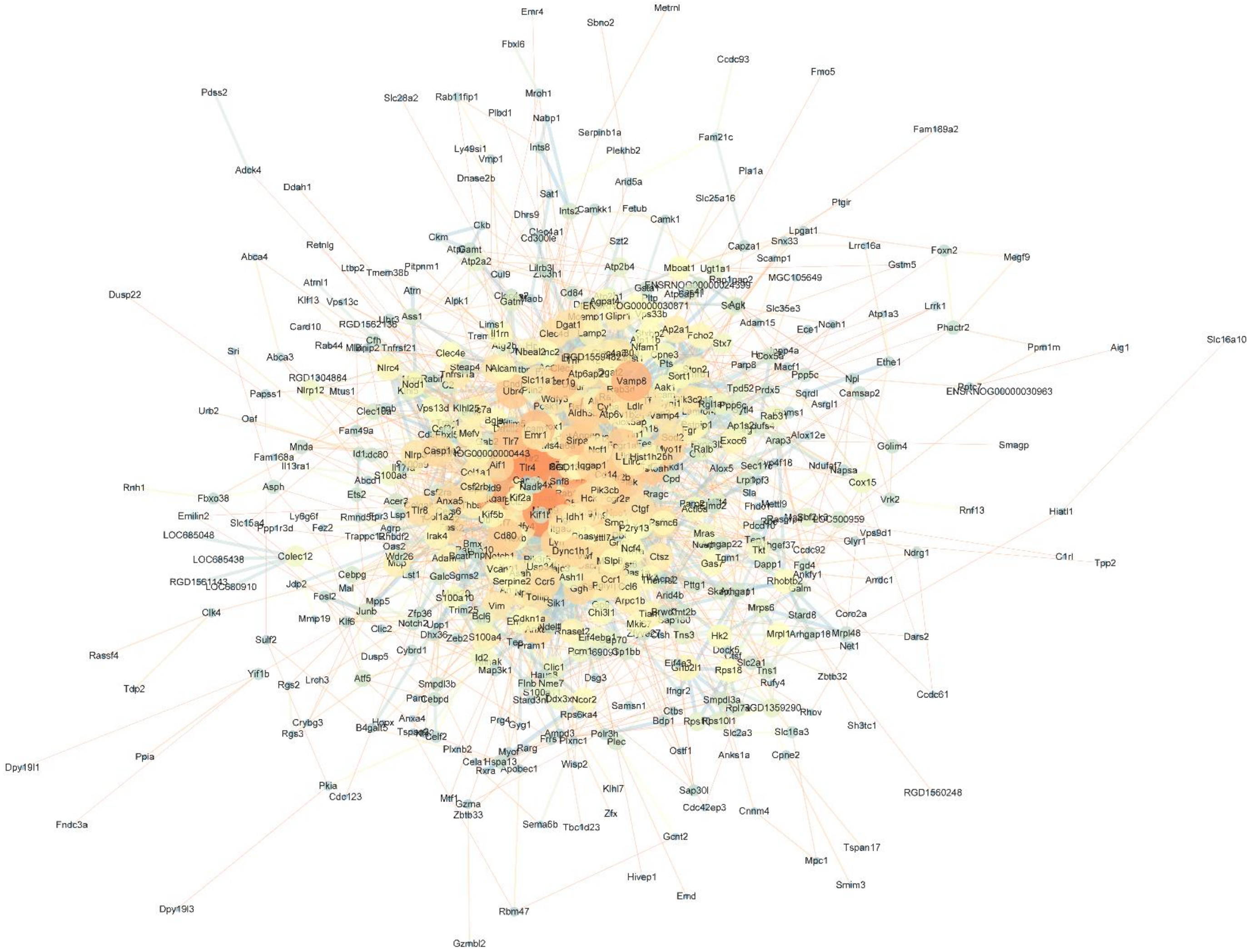

Supplemental Figure 2: Network visualization for the protein-protein interaction (PPI) network of all potential target genes from transcriptome analysis. The data of PPI was from STRING and the PPI was made by Cytoscape. The protein without any node is deleted. The larger the circle, the more adjacent nodes. Average number of neighbors is 9.025. There are 189 genes with number of neighbors greater than 9.025.
